# Programme theory and linked intervention strategy for large-scale change to improve hospital care in a low and middle-income country - A Study Pre-Protocol

**DOI:** 10.12688/wellcomeopenres.16379.2

**Published:** 2020-12-18

**Authors:** Mike English, Jacinta Nzinga, Grace Irimu, David Gathara, Jalemba Aluvaala, Jacob McKnight, Geoffrey Wong, Sassy Molyneux

**Affiliations:** 1 Health Services Unit, KEMRI-Wellcome Programme, Nairobi, Kenya; 2 Centre for Tropical Medicine and Global Health, University of Oxford, Oxford, UK; 3 Nuffield Department of Primary Care Health Sciences, University of Oxford, Oxford, UK

**Keywords:** Quality Improvement, Health Systems, Neonatal Care, Evaluation, Hospital

## Abstract

In low and middle-income countries (LMIC) general hospitals are important for delivering some key acute care services. Neonatal care is emblematic of these acute services as averting deaths requires skilled care over many days from multiple professionals with at least basic equipment. However, hospital care is often of poor quality and large-scale change is needed to improve outcomes. In this manuscript we aim to show how we have drawn upon our understanding of contexts of care in Kenyan general hospital NBUs, and on social and behavioural theories that offer potential mechanisms of change in these settings, to develop an initial programme theory guiding a large scale change intervention to improve neonatal care and outcomes.  Our programme theory is an expression of our assumptions about what actions will be both useful and feasible.  It incorporates a recognition of our strengths and limitations as a research-practitioner partnership to influence change. The steps we employ represent the initial programme theory development phase commonly undertaken in many Realist Evaluations. However, unlike many Realist Evaluations that develop initial programme theories focused on pre-existing interventions or programmes, our programme theory informs the design of a new intervention that we plan to execute. Within this paper we articulate briefly how we propose to operationalise this new intervention. Finally, we outline the quantitative and qualitative research activities that we will use to address specific questions related to the delivery and effects of this new intervention, discussing some of the challenges of such study designs. We intend that this research on the intervention will inform future efforts to revise the programme theory and yield transferable learning.

## Introduction

All health systems are striving to improve service quality. In low and middle-income countries (LMIC) this is essential if efforts to enhance access through universal coverage are to deliver better health outcomes ^1^. General hospitals in LMIC are especially important for delivering services that cannot feasibly be provided in the community or primary care clinics and do not require tertiary care expertise ^2^. Neonatal care is emblematic of hospital care as averting many deaths requires skilled professionals and at least basic equipment while care is delivered consistently and carefully over many days and nights ^3^. Currently however, research suggests such care is poor ^4, 5^. As many LMIC are now hoping to scale up essential hospital based neonatal care ^6^, understanding how to change and improve services at scale is urgently needed. The enhancements in team-based care to provide respiratory support, patient monitoring, infection control and many other aspects that are central to good neonatal outcomes are also critical to scaling up access to other forms of acute hospital care, including severe coronavirus disease (COVID). Lessons from large scale change in one sphere may therefore have much wider value.

Emphasising that these learning needs are not specific to LMIC, the NHS Institute for Innovation and Improvement recently focused attention on this challenge and suggested large scale change interventions are ^7^:

‘ *widely spread across geographical boundaries, multiple organisations, or multiple distinctive, groupings (e.g. doctors, nurses, managers, social care workers), deeply challenging to current mental models and ways of thinking (it feels uncomfortable and evokes some push-back from others because it is so different from the usual), broadly impacting on what people do in their lives or time at work and requiring co-ordinated change in multiple systems.’*

This definition comprises three broad change dimensions; size / scale (spanning both geography and actor groups), depth (of cognitive / behavioural shift), and pervasiveness (whether it affects the whole or part of a system). The implication is that careful thought is needed on the nature and magnitude of challenges that may need to be overcome with respect to these dimensions if an intervention strategy is to be successful. This definition also emphasizes that it is people, as individuals, groups and organisations, not things that are a primary focus of change strategies. So, while we must pay attention to technical elements of care a major part of our intervention thinking must be focused on how we change individual and collective behaviours. Importantly, this demands intervention at multiple levels in a system, and therefore consideration of the multiplicity of potential interactions between different actors ^8^. This requirement distinguishes large scale change thinking from many implementation interventions that focus, for example, on introducing a specific clinical guideline or technology into a particular work context. Studies on large scale change interventions to improve service delivery have been identified as a particular gap in existing LMIC research ^1^.

Our long-term aim is to design, deliver and evaluate a large-scale change intervention targeting improvement in neonatal care in Kenyan general hospitals and in so doing develop transferable lessons to guide future large-scale change efforts targeting hospital care. Our focus is on hospitals that do not offer tertiary care and that are not large regional specialist centres. They may aspire to offer an intermediate level of care ^9^ and are often called district hospitals in LMIC (or county hospitals in Kenya). These hospitals often have between 80 to 300 inpatient beds in total, serve populations of 100,000 people or more and are by far the most numerous in LMIC. Here we use the term general hospitals to indicate their broad, non-tertiary status.

In this manuscript we aim to show how we have designed an intervention and linked evaluation focused on improving outcomes in Kenyan general hospital NBUs. To do this we used our understanding of contexts and what we feel are relevant social and behavioural theories that offer potential mechanisms through which change might be achieved. Contexts here refers to (for example) the characteristics of the hospitals as organisations, the human and material resources that are available within them and also the existing cultures and norms that influence practices. We introduce theories that span multiple levels of a health system. We focus on those that seem to us the most pertinent guides on how to modify these contexts so that they trigger the social and behavioural mechanisms we suggest would be effective at improving the provision of care and outcomes at scale. We go on to acknowledge how our selection of theories and intervention strategies is necessarily influenced by our capacity and positionality as a researcher – practitioner partnership. The fact that we have neither the formal authority or resources of government precludes us proposing major changes to the resource or formal organisational contexts in which care is taking place. We use a reflective and iterative process to draw together these interlinked strands of understanding spanning clinical practice, context, potential mechanisms of effect and the capacity of our researcher – practitioner partnership. This reflective and iterative process helps us synthesise our insights to develop an initial programme theory and design our intervention. The programme theory is, therefore, an expression of our assumptions about what actions will be both useful and feasible when seeking to address the challenges we have identified in neonatal care and achieve improvements in care and outcomes.

The steps we employ represent the initial programme theory development phase commonly undertaken in many Realist Evaluations ^10^. However, unlike many Realist Evaluations in which researchers study interventions or programmes that have been designed and often are already being delivered by other parties, we use this phase to design a new intervention we also plan to execute. Subsequently, therefore we articulate briefly how we propose to operationalise the intervention. Finally, we outline the quantitative and qualitative research activities that we will use to address specific questions related to the delivery and effects of this intervention (phase two of realist evaluation) that will inform future efforts to revise the programme theory (phase three of realist evaluation).

## Neonatal care and key outcomes in Kenyan and LMIC hospitals

The need to reduce neonatal mortality (deaths in the first one month of life) across LMIC has risen to prominence as neonatal deaths now account for 45% or more of all mortality under five years of age ^11^. Reducing neonatal mortality by 2030 is therefore a specific Sustainable Development Goal target (SDG 3.2) ^12^. Achieving this will require the transformation of poorly functioning general hospital NBUs ^3^. Currently mortality on hospital NBUs is high in most LMIC, often 20-fold higher for some conditions than in high-income countries, and Kenya is no exception ^13^. As well as providing medical interventions for sick babies (e.g. advanced respiratory support, oxygen, antibiotics and intravenous fluids) care must include, amongst many other facets, careful monitoring, infection prevention and initiation of enteral feeding. All the while teams must share information and provide emotional support to parents so they bond with their baby and engage in providing care. In many respects the clinical needs of sick newborns are therefore similar to those of all age groups who have severe, acute illness including those with moderate and severe COVID-19 ^14^.

**Figure 1.**
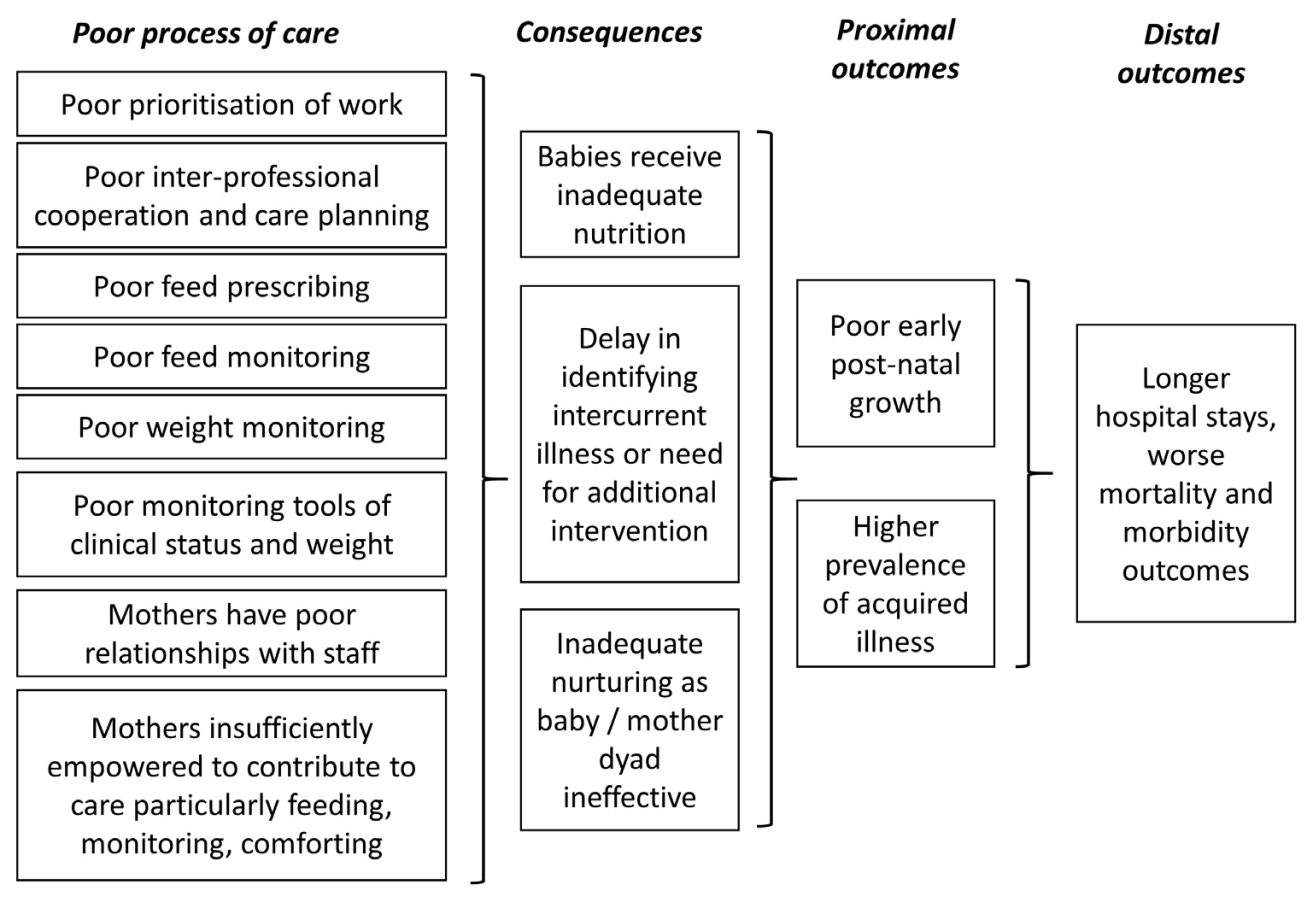
Simple driver diagram outlining the links between poor quality process of care and poor proximate and distal outcomes. The drivers on the left can collectively contribute to immediate consequences and then proximal and distal health outcomes.

Mortality rates are a potentially useful metric of the success of care, although many caution against their use as measures of quality ^15^. An additional outcome spanning many of the interacting elements in care that is more specific to the newborn population is achieving adequate initial nutrition and thus early weight gain. This is particularly true for those born preterm or with a low birthweight for whom health and weight gain are closely related. Our initial experience and data point to high mortality and suggest that babies’ clinical monitoring, growth and nutrition are given insufficient attention in Kenyan NBUs ^13^ (unpublished data). At the same time mothers are given little educational or emotional support, reducing their ability to engage productively in their baby’s care ^16^. These gaps in care put babies at continued risk in the short term and may also threaten babies’ long-term development ^17^. We summarise some of these gaps in care in an example driver diagram that points to their proximate and distal consequences ( Figure 1). While data are limited on quality of care from LMIC NBUs, we believe the situation in Kenya has much in common with many other countries ^18, 19^. Intervention strategies that can improve multiple facets of care on NBUs or in clinical areas facing similar challenges in LMIC hospitals therefore seem urgently needed ^20^.

One major challenge in many LMIC including Kenya is that hospital management and monitoring systems are weak and there are major human and material resource constraints ^13, 21^. These challenges affect hospitals’ delivery of inpatient maternal, surgical and adult medical care as well as paediatric and neonatal care ^22– 24^. This means there is very limited organisational and resource slack to mobilise for any new purpose. Interventions that seek to achieve large scale change must therefore either consider how to mobilise new resources or consider what is achievable with limited resources.

LMIC health systems perhaps also differ in their organisational structure from higher income settings. Employing Mintzburg’s characterisation of organisations Blaise and Kegels suggest LMIC health systems are predominantly organised in a ‘command and control’ fashion in contrast to the professional bureaucracies of high income countries’ (HIC) health systems ^25^. However, in some LMIC including Kenya, devolution for operational aspects of health service delivery to local administrations is considerably weakening the potential for national command and control mechanisms to effect change at scale ^26^. In HIC the impact of top-down efforts to implement change depends heavily on the mediating role of senior professionals individually and collectively ^27^. In many African settings such as Kenya, however, the potential mediating role of senior medical professionals and their historically younger associations have rarely been explored. Indeed, such senior professionals have only recently been consistently present in larger general public hospitals outside major cities in many LMIC. For example, Kenya has fewer than 100 specialist paediatricians working in general public-sector hospitals but these facilities provide the majority of hospital care to its population of over 50 million ^28^. However, even having only one or two paediatricians leading large and busy general service units is a relatively recent phenomenon. Working with such senior professionals in ways that have proven productive in HIC are now therefore an emerging possibility ^29, 30^.

When senior professionals are very few the consequence is that junior physicians, non-physician clinicians and clinician trainees provide most of the ward based clinical care ^31^. Such junior medical staff often rotate regularly through different departments. As a result, they may not develop significant expertise in more specialist units such as NBUs where they may spend only a few weeks. In many hospital settings, therefore, the institutional memory, organisational culture and practical norms that frame service delivery may largely be vested in senior hospital nursing staff ^32^. This makes them an influential but largely neglected group of hospital practitioners. Such nurses very rarely have specific specialty training (e.g. in neonatal or critical care nursing) and have not traditionally been part of specific professional nursing networks in countries such as Kenya.

What are the problems in delivering quality care in Kenyan NBU? In prior work in Kenya we identified significant material and human resource limitations ^21^. Often, for example, a single nurse is responsible for the care of 15 or more babies on a NBU or paediatric ward ^33^. To maintain a sense of order, nurses adopt routines to structure their NBU work that may in some cases be detrimental to achieving good patient outcomes while there is relatively poor inter-professional cooperation ^32, 34^. Perhaps most problematic are data that underpin our driver diagram ( Figure 1) suggesting that many important aspects of care, including for example regular feeding, may simply be missed or informally shared with untrained staff or mothers ^33, 35^. There is typically little evidence that NBU teams collectively focus on achieving the high priority tasks and important care goals outlined in Figure 1. What is clear is that any large-scale change strategy will need to engage both medical and nursing staff.

Although the initial conditions we outline do not seem promising ground on which to launch improvement initiatives, previous work suggests some change can be achieved at scale in similar settings ^36, 37^. Favourable conditions include the existence of widely accepted and disseminated common practice guidelines ^38^ that are reinforced by short training programs in medical schools and attended by many young physicians ^39, 40^. More recent efforts have been to build consensus around improved nursing care ^41^.

Supplementing our own experience, there are reports of efforts to achieve large-scale change in other LMIC. These include some successful programmes in Papua New Guinea, Ghana and Rwanda amongst others ^42– 45^. Some of these have employed extensive quality improvement approaches based on the model of collaboratives ^46, 47^. However, there have been fewer efforts to elaborate how these hospital focused LMIC programmes have been informed by theory. In some of our earlier work that aimed to change paediatric ward practices in Kenya we drew predominantly on behavioural implementation science models ^48, 49^. This prior work helped foster the small-scale development of a network that links together general hospital paediatricians ^50^. This experience prompted us to explore a broader range of theories relevant to large scale change.

## Drawing on theory and potential opportunities for large scale change in hospital care

**Figure 2.**
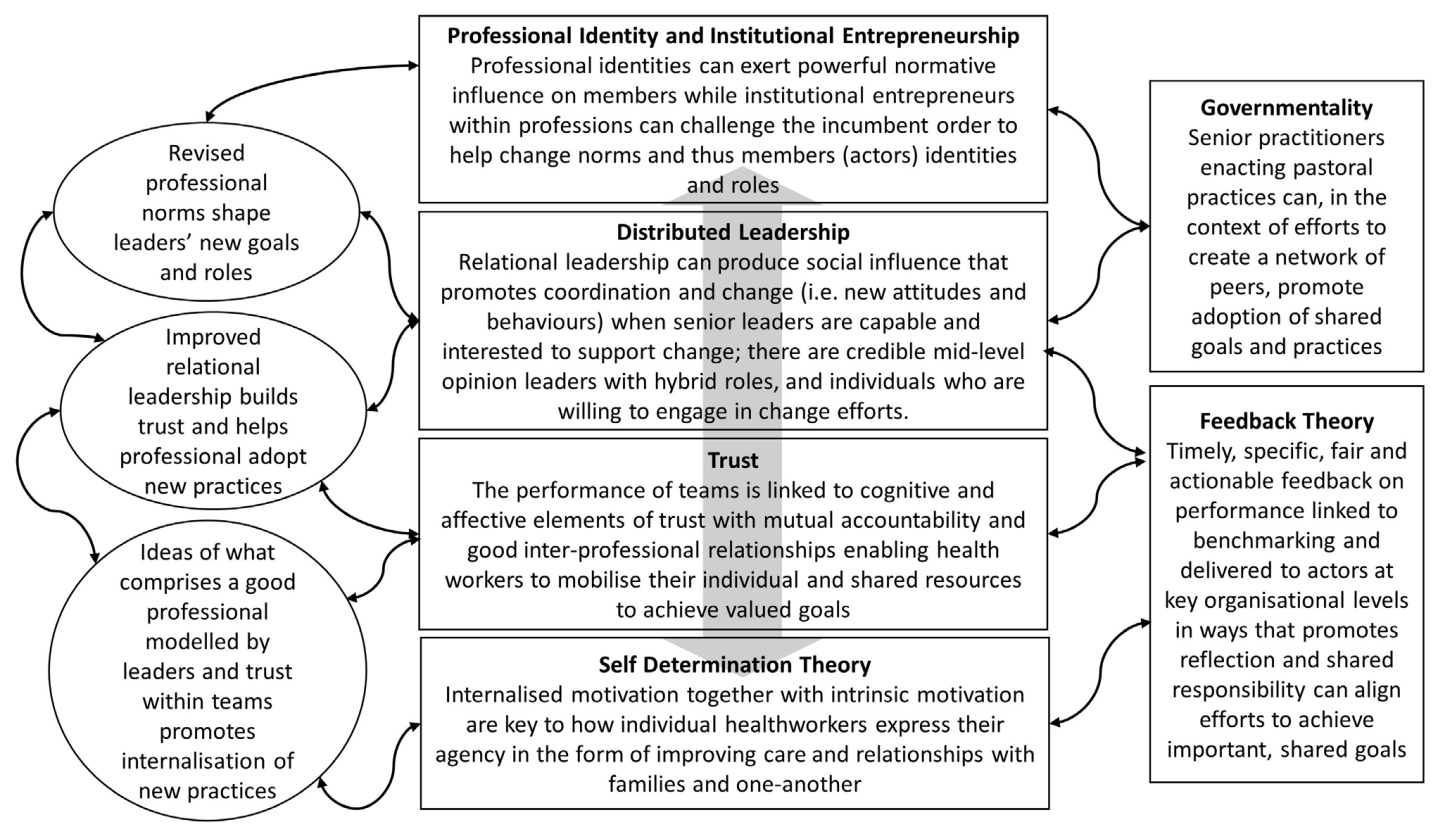
Summary indicating the layers and nature of theory we draw on in our thinking about large scale hospital change in low and middle-income countries. We draw on the figurative portrayal employed by Westhorp ^51^ and represent our main influences in the central column and additional influencing theories in the right-hand column. In the left-hand column we indicate some of the effects we hope to see.

Our aim is to base intervention strategies on an understanding of how things work and so to propose mechanisms that can later be evaluated. When employing theory to do this we draw on the work of Westhorp who suggests using different layers of theory as ‘both complexity theory and a realist philosophy of science understand reality as comprising multiple, nested, open systems in which change is generative, context dependent and time irreversible ^51^.’ Pawson and Tilley in their use of Realistic Evaluation focus on the ability of different contexts to trigger mechanisms that will produce outcomes ^10^. We therefore explored theories that would help us anticipate how an intervention might result in change at different levels of the health system and in particular change the responses of multiple system actors to produce desired outcomes ( Figure 2) ^51^. Given our prior work we were also interested in the potential of networks as vehicles to deliver such change interventions at large scale.

### Networks as a vehicle for delivering change

Three decades ago Powell ^52^ characterised networks as

*‘non-market, non-hierarchical modes of exchange [that] represent a particular form of collective action in which: i) Cooperation can be sustained over the long run as an effective arrangement; ii) Networks create incentives for learning and the dissemination of information, thus allowing ideas to be translated into action quickly; iii) The open-ended quality of networks is most useful when resources are variable and the environment uncertain; iv) Networks offer a highly feasible means of utilizing and enhancing such intangible assets as tacit knowledge and technological innovation’.*

In health, networks exist in multiple forms, spanning fully integrated service delivery systems to informal communities of practice. Recent interest in the context of service improvement has included networks characterised by ‘voluntary clinician groupings that aim to improve clinical care and service delivery using a collegial approach to identify and implement a range of strategies’ ^53^. Important points for us here are shared goals and the absence of direct financial incentives to network members that seem especially relevant for public sector organisations. Also, at least within high income health settings, it has been reported that ‘important ingredients for successful clinical networks were visionary and strategic leadership with strong links to external stakeholders; and having formal infrastructure and processes to enable the development and management of work plans aligned with health priorities’ ^54^. Others have pointed to the potential of networks to link those seeking knowledge or information to others with such information ^55^ and recently ‘non-hierarchical collaborative networks’ have been highlighted as a useful element of system interventions in LMIC ^56^. Networks have also been proposed as having roles in tackling wicked problems inherent to the complexity of health care organisations ^29^ and as a model for implementing normative governance that does not rely on formal regulation ^57, 58^.

Networks are, however, inherently a form of complex system. The characteristics of complex (adaptive) systems are, amongst others: i) that they are open systems (so that even if your research has boundaries there will always be influences arising from beyond these boundaries), ii) they have agents whose interactions cannot always be predicted or controlled, and iii) they adapt, interact and co-evolve with other systems ^59^. Here we take agency to be defined ‘as the cognitive, motivational and emotionally driven behaviours that agents employ to achieve their end goal’ ^60^. As a result of these properties, complex systems can behave in ways that are not entirely predictable. This suggests that attention must be paid to design the process of intervention enabling flexibility while ‘keeping things on the rails’ with respect to its initial principles.

### Professional identity and institutional entrepreneurship

We seek to change care practices in multiple hospitals. Hospitals are formally comprised of people organised within multiple hierarchies but they are also highly professionalised and governed as much by customs and values as rules and regulations. Together these influences ‘shape the rules of the game’ operating in hospitals ^61^. We recognise that senior professionals may be subject to ways of thinking linked to their profession (as doctor or nurse) and sometimes to hybrid roles they may have as managers ^62, 63^. These ways of thinking, or institutional norms, may conflict between professions, or within individuals with multiple roles, and some institutional norms may dominate in different times or settings. Of relevance to our intervention we seek to ‘provide internal reformers with arguments for change’, and ‘provide them with the community and solidarity necessary to take risks’ ^64^. What we hope to foster therefore, are institutional entrepreneurs so that change may be brought about by professionals collectively setting a new agenda and then individually acting out this new agenda ^30, 65, 66^.

If such professionals, as institutional entrepreneurs, are collectively to succeed then their emerging professional bodies will need to become engaged as important institutional champions of improved care. Professional associations have been the subject of little formal attention in LMIC. We suggest the identity professional bodies currently give shape to, like their antecedents in HIC, are much more closely aligned with that of expert medical practitioner than of service manager striving for local improvement ^63^. Moreover, in Kenya professional bodies may be dominated by members from the private sector, for example less than 20% of the Kenya Paediatric Association membership practice in public sector hospitals below the tertiary level ^28^. Our interventions may then also need to try and shape such bodies’ roles as advocates at the highest level so they are better aligned with the specific needs of those serving public sector improvement goals.

The movement of specialist clinicians into general hospitals in countries like Kenya is occurring within the context of existing organisational hierarchies. Doctors are often thought to be at the apex of the healthcare professional hierarchy while nurses, typically providing more holistic patient-focused care, are often considered subordinate to doctors ^30, 67^. However, in the case of more specialised units such as neonatal wards, senior nurses may have an elevated status resulting from long experience and exert considerable autonomy and authority within this microenvironment ^68^. Both professional groups, doctors and nurses, are therefore critical to transforming care in NBU and, we argue, in LMIC hospitals more widely.

The potential value of a network strategy is that it may foster emergence of local institutional entrepreneurs, may support their growth in these roles by helping shape the identity of professional bodies, may enable different professional groups to find common purpose and may thus promote behaviour change at scale ^29, 58^. Should professionals take on these newer roles then they must also provide leadership.

### Distributed leadership

Distributed leadership has been characterised by Fitzgerald ^69^ as comprising three components: senior leaders with the capability and interest to support change; credible opinion leaders at middle levels who hold hybrid roles; and individuals who are willing to engage in change efforts through a social influence process ^70^. It is a particularly useful conceptualisation of leadership for the public sector, multi-professional general hospitals which are the settings of focus ^71^. Within a network, influences from peer groups, professional associations, and the external intervention team may all reinforce NBU leaders’ sense of accountability for service improvements. A key role for NBU leaders is then to support front-line workers to change ^72^. To do this, they will need to set a local strategic direction or vision and develop a culture of continuous improvement on the NBU ^73^. Good relational skills may help NBU leaders promote changes in practices and they must therefore be good communicators and able to create trust amongst the NBU team members. Failure to do this may undermine the intervention’s change effects ^27^.

### Trust and teams

Trust has political, organisational and social dimensions that are important to the entire health system ^74^. Here we are especially concerned with trust between people, and especially trust within teams that deliver everyday care on NBU. In inter-personal relations trust may be thought of as having two components, cognitive and affective trust ^75^. In the former, individuals look for a rational reason to trust the other party. Considered in this way individuals take the risk that those they trust will act in ways they can anticipate, that are fair and that do not make them unduly vulnerable ^74^. Affective trust is linked more to notions that those trusting and those trusted have a mutual, emotional investment in a relationship ^75^. This may result from shared experiences or values. Maintaining affective trust may be especially important in the high stress, resource limited environments of LMIC hospitals and help sustain ‘everyday resilience’ ^76^.

A team can be defined as two or more people who interact and are mutually accountable for achieving common goals and who perceive themselves as a social entity within an organization. Their combined cognitive resources and skills should help them outperform individuals especially in complex and dynamic environments such as hospitals. Teams often already have, however, long-established ways of working. To change these ways of working, team leaders need to be trusted by team members and demonstrate that they have the competence to lead, reflecting both task and relationship oriented skills while showing benevolence and integrity ^77^. Further, they should demonstrate supportive, participative, and empowering leadership behaviours, indicating that they have confidence in, and concern and respect for, their team members ^78, 79^. Interventions that can enable multiple team leaders to learn these skills, communicate well and engage team members in mutually trusting relationships may therefore be key to improving care at scale.

### Feedback

Measurements of the process and outcomes of care used in performance monitoring may have beneficial and sometimes harmful system effects ^80^. Theories suggest that for measurement to be effectively used feedback to those whose performance is being assessed should be based on trusted data and be timely, specific, non-punitive and customisable while also providing clear goal oriented advice on what actions may be needed to improve ^81– 83^. When trying to promote the effects of feedback to teams we need to be aware that this will be influenced by their interactions, communication, and individual perceptions of the feedback ^84^. Furthermore, individuals given team-level performance information have to figure out to what degree they feel these data reflect their individual input ^85^. In theory, feedback based on the work of the whole team can promote cohesion, co-operative work and shared goals ^85^. Alternatively, individuals can decide that others and not them are responsible for poor performance, making feedback ineffective or potentially harmful. How feedback is perceived may depend on it being linked to strategies that promote shared reflection on what the feedback means and what the team needs to do in response ^86^. Indeed, what may be important is the notion of ‘feed-forward’ ^87, 88^, which suggests that it is reflection on progress towards a shared standard or goal and how to close performance gaps that may be most useful. The benefits of feedback may thus rely on the ability of an intervention approach to create widely shared ideas about ‘good performance’ and link this to mechanisms for shared reflection and problem solving at local levels. Re-shaping institutional norms linked to effective leadership behaviours of network and local actors that inspire trust may be central to efforts to do things differently.

### Motivation and self-determination theory

Health workers must have the motivation to improve care. Michie and colleagues provide a useful framework of influences on individual behaviour that centre on capability, opportunity and motivation ^89^. In this model, capability encompasses both physical and psychological capacity to act in a desired way and thus includes having relevant knowledge and skills. Opportunity is defined as all those factors that lie outside the individual that make the behaviour possible or prompt it. It thus includes physical factors (e.g. job aides) and factors such as the expectation of peers and leaders that are part of people’s social environment that influence their behaviour. Ideally an intervention might help develop the capability and opportunity of teams by enhancing the working and wider professional environment that supports change. Motivation in this model also has two aspects. One involves reflective processes, how do the actions required as part of the change align with personal plans and goals, the expectations of others and their trust in the way we behave. The second is considered more directly aligned with emotions and automatic responses. The latter has similarity with the notion of intrinsic motivation when a behaviour is naturally satisfying – it brings its own rewards. In this case it may help if desired behaviours are aligned with health workers’ sense of altruism as even if the actor has no obvious gain the behaviour may be adopted ^90^.

Extrinsic motivation in contrast refers to situations when a behaviour is linked with an external reward (pay-for-performance is a topical example) ^90^. Some external rewards focused on increasing extrinsic motivation may decrease intrinsic motivation, a situation sometimes referred to as ‘crowding out’ ^91^. This may, for example, help explain the failure of many pay for performance initiatives ^92^. Importantly, the focus on monitoring as part of performance management linked to sanctions or rewards that characterises New Public Management may also crowd out intrinsic motivation. This may worsen performance in areas of the public sector that are traditionally associated with a service or vocational ethos such as health care ^91, 93^. Thus, efforts to improve quality of care that include monitoring and performance feedback might backfire if they are regarded as a form of externally imposed surveillance that undermines feelings of competence (being a better professional) and autonomy (a willing choice to adopt new behaviours) at work that are important to professionals ^90^. To avoid this, it is important that feelings of competence and autonomy are enhanced as part of change efforts, both may be facilitated by the process of fostering active participation in and ownership of the intervention and carefully framed feedback.

Further insights on motivation can be gleaned from self-determination theory. This has at its core a distinction between autonomous motivation and controlled motivation. Intrinsic motivation is a form of autonomous motivation while controlled motivation reflects a sense of acting under pressure, of having to engage in actions ^90^. An important contribution of this theory is that it suggests that extrinsic motivation can vary in the degree to which it is autonomous or controlled (rather than maintaining the simple dichotomy of intrinsic and extrinsic motivation). It proposes that behaviours that are not intrinsically motivating can be externally regulated, that is initiated and maintained by contingencies external to the person. However, self-determination theory posits that some external regulatory influences can become internalised. Internalisation is defined as ‘people taking in the [desired] values, attitudes or regulatory structures’, such that the external regulation of a behaviour is transformed into internal regulation and no longer requires the presence of the external contingency (or condition) to sustain a desired behaviour ^90^. An example of this might be the adoption of evidence-based practices. Initially the standardisation of care linked to evidence based practice was seen to limit professional autonomy and was often rejected ^94^, but sustained external regulatory forces may now have resulted in many professionals internalising the idea that guidelines are important (they have value) and that following them reflects good professional practice ^58, 73^. Thus, while there may be no satisfaction in adhering to best practices - they are not intrinsically motivating – their use is reinforced by a mindset (or mindline ^95^) that equates them with better care (internal regulation). The aim of most efforts to scale up improved health care provider practices, and the ultimate focus of this proposal, is to make best practices routine. To do this we hope to achieve what self-determination theory refers to as integrated regulation, when ‘people have a full sense that the behaviour is an integral part of who they are…..that it is thus self-determined’ ^90^. From a broader organisational perspective this process is encompassed in ideas of governmentality ^58, 96^. This goal aligns with wider efforts to promote behaviours that improve care as part of changes in health workers’ professional identity.

## An initial programme theory for an intervention targeting improvements in care on Kenyan NBU

Above we have suggested that a network strategy may foster emergence of local institutional entrepreneurs, may help shape the identity of professional bodies, and may enable different professional groups to find common purpose to promote change at scale. The NBU leaders who might take on day to day change roles might then support front-line workers to change. To achieve this, there will need to be shared goals and the leaders must foster a culture of continuous improvement. For this, leaders will require good relational skills so they can communicate well with team members and develop mutually trusting relationships. Feedback on progress linked to locally shared goals and mechanisms for shared reflection and problem solving within teams that trust one another at local levels may motivate change and promote the adoption of new and better practices that come to be seen as ‘part of who we are’. Paying attention to improving the day to day tools people use through co-design so they enable better work may have direct benefits on care processes and wider benefits in fostering shared ownership of problems and their solutions. Through this multi-level change strategy, we expect to be able to address the key drivers of poor care outlined in Figure 1. For example, co-designed tools may help improve feed prescribing, and feed, clinical status and weight monitoring. Those institutional entrepreneurs who adopt leadership roles to drive use of these new tools may be encouraged in their efforts by feedback and if team communications are improved. The better monitoring resulting may improve babies’ nutritional intake and allow earlier identification of intercurrent illness. Similarly, greater professional recognition of the needs of families, more effective use of audit in a safe (trusted) space that allows discussion of respect shown for families, and improved communication amongst staff and between staff and families may empower more active engagement of families in care. This too may lead to improvements in intervention delivery (eg. feeding practices) that benefits babies’ health. Thus, as interventions may address particular clinical concerns the reshaping of the social and organisational context achieved by the set of network activities may be key to their success. In planning and running such multi-level change efforts considerable attention must, therefore, be paid to how these intervention processes are taking place within a complex system. In such a system it is hard to isolate specific, linear causal pathways between an intervention and a target care process.

In higher income countries large scale efforts to improve care may be driven by a political agenda, in response to poor performance assessed by government or other payers, or user dissatisfaction. In some cases, they may be driven directly by powerful professional groups. Government initiatives in HIC may attract significant direct financing and leverage considerable indirect support offered by existing infrastructure or organisational arrangements (e.g. by using established performance measurement systems or co-opting local quality improvement teams). There are few such resources in LMIC generally or in Kenya specifically so our change strategy must be tailored to the resources available and the position within the system of those aiming to intervene.

**Figure 3.**
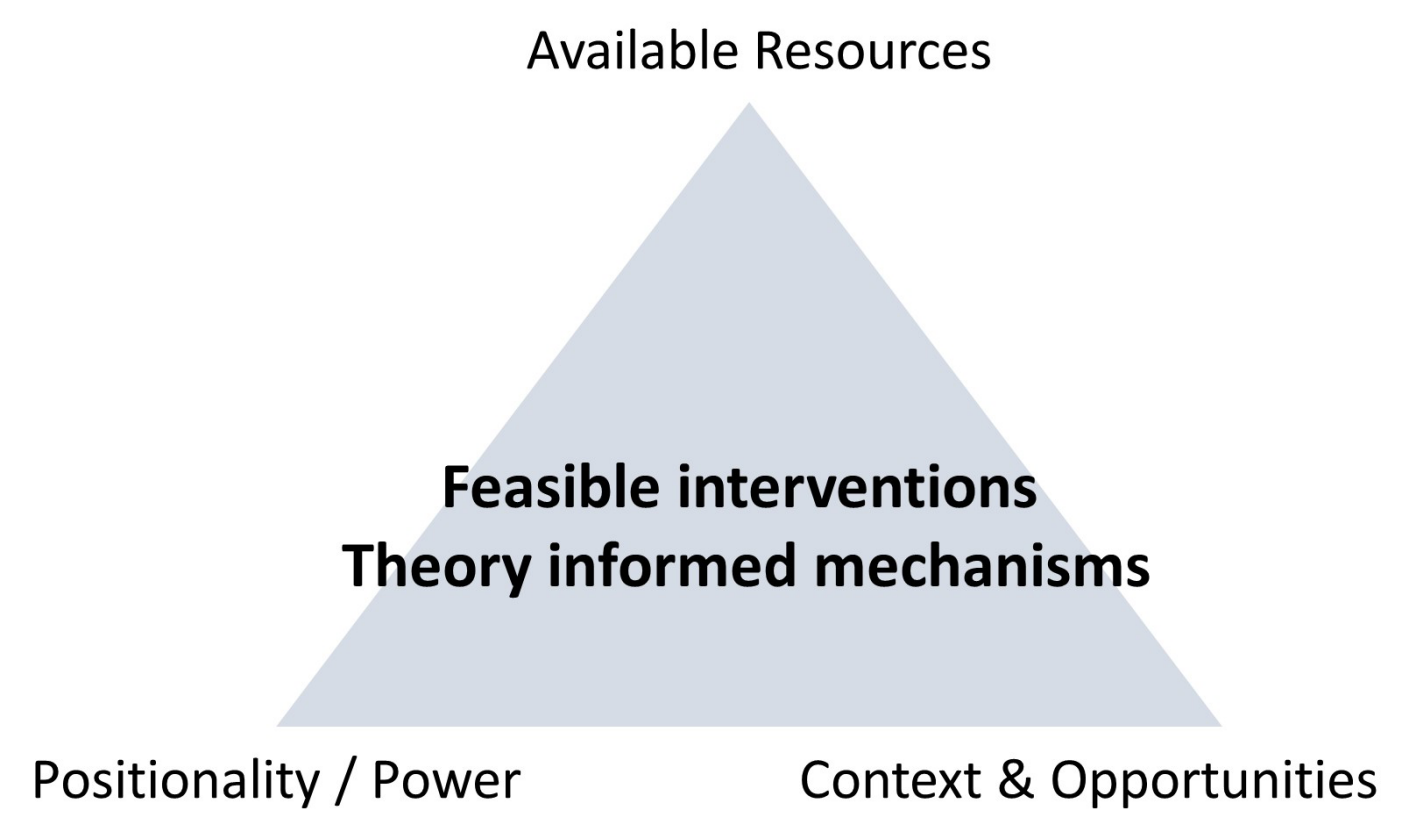
Anchors in our reflective and iterative process that guided design of the specific Kenyan newborn unit intervention strategy. In this depiction we illustrate the major thematic factors we continuously navigated between as we sought, through an iterative and reflective process involving the authors and multiple team members, to link our understanding of context to potentially feasible intervention strategies aligned with our understanding of theories that offer potential mechanisms through which change is achieved.

Our proposed change efforts, however, do not start from a blank slate. All health systems have a history that defines them as starting contexts and this introduces elements of path dependency. We had already established a clinical information network that focused on paediatricians, use of common data and adoption of guidelines on paediatric hospital wards ^50^. This Clinical Information Network included stakeholders from the research community, the paediatric professional association, hospitals’ paediatric teams and the government. Extending this to tackle improvements in NBU care seemed an obvious starting point and gives us an awareness of the resources we might directly mobilise to support change. At the same time, we must constantly consider our position as a research team with no formal role or authority in planning, managing, providing or governing health care delivery. In these regards, our position is perhaps similar to that of many non-governmental partners supporting LMIC health systems. Key to this, and building on the insights outlined earlier this meant: i) defining a clear neonatal clinical focus and desired outcomes, ii) defining the boundaries of the system we aim to influence by focusing on general hospital NBU, and iii) using our knowledge to identify issues we feel are within our power to influence as an embedded research team. In continuously returning to the bodies of theory outlined above we also tried to maintain a focus on the mechanisms through which intervention components might work, their potential interactions, and the outcomes that might be achieved ( Figure 3).

**Figure 4.**
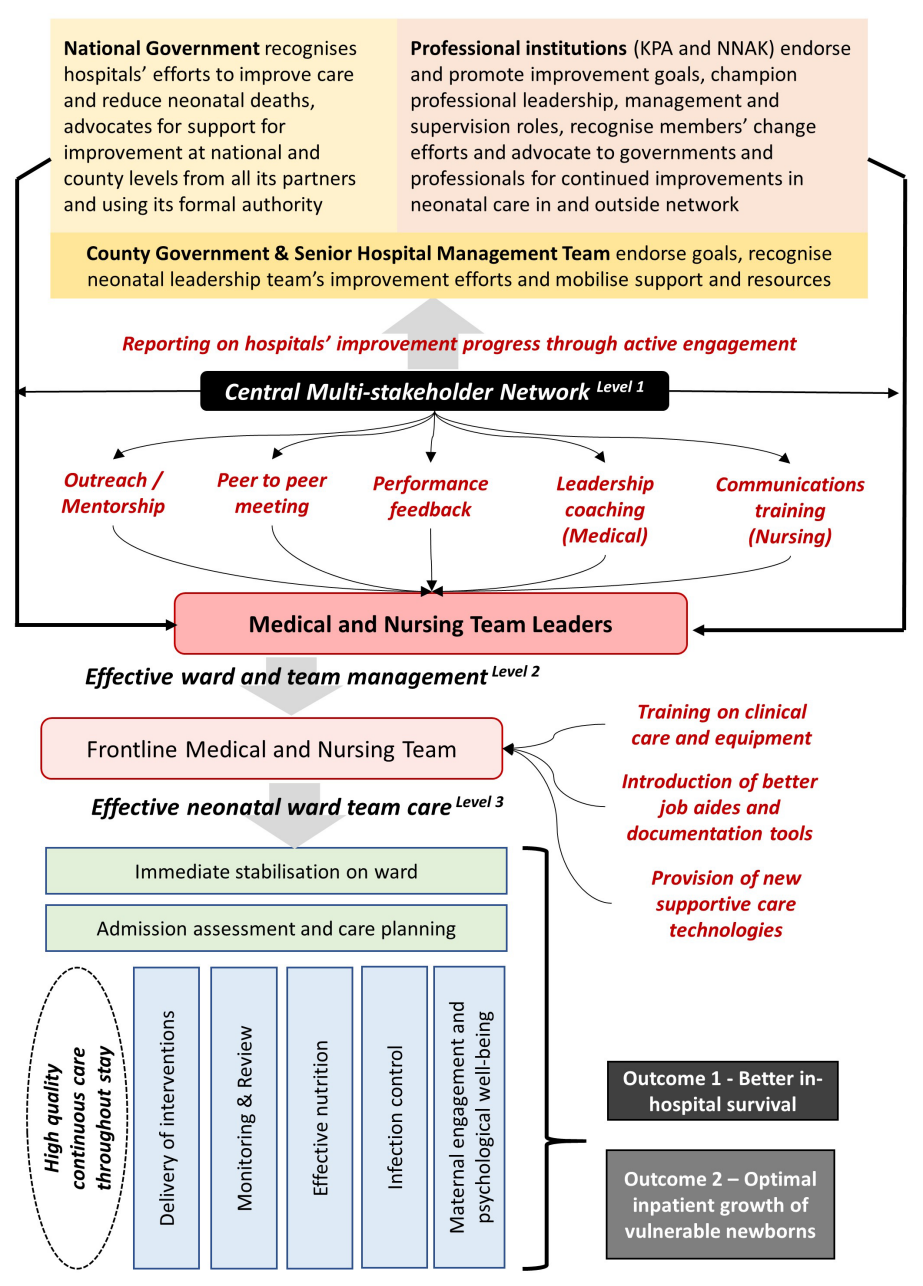
Programme Theory for a Network Intervention to achieve large scale change across multiple Kenya hospitals. The central network operates at the interface between national stakeholders and hospitals, what we consider level 1 in this network system. It bears responsibility for engaging with and providing timely performance information to key departments in the national and county governments and to the paediatric and nursing professional associations. At the same time this central team is responsible for providing hospitals with feedback on performance using quality indicators and working with NBU leaders to create and build a peer-to-peer network that includes face-to-face meetings, and provide expert outreach using a mentorship model, and co-opting additional resource persons to support the development of leadership, management and communication skills of NBU leaders and teams. The selection of these intervention components reflecting identified challenges and prior experience of successful intervention ^16, 33, 45, 48, 58, 63, 71, 76, 98^. The NBU leaders operate at what we consider to be level 2 in the network system, interfacing with the central network but also their hospital specific NBU teams and senior management. At level 3 in this system are the frontline workers led by their team leaders who are the critical interface with the sick newborns themselves and their families. The Central Multi-Professional Network team members may have little direct contact with or influence on events at level 3, any network effects will therefore predominantly be mediated by those at level 2. The paediatricians and senior nurses at this critical level 2 juncture, who operate at the mid-level of the hospital’s management structure, have rarely been prepared for their leadership roles or given any specific support to build the relational skills that are likely critical ^63, 71, 98^. Enhancing the capability and motivation of these level 2 individuals is therefore a key aim of the network intervention.

Our overall Programme Theory is presented in Figure 4. It draws generally on the theory and contextual influences outlined above but we use the structure of ‘If, then, because’ statements to propose much more specific requirements that should be achieved, assumptions that can then be examined in subsequent research ( Table 1) ^97^. At its heart is a central Multi-Professional Network. Operationally we aim that this will comprise two to three respected paediatricians (part-time) and a respected senior nurse (full-time) supported by a small team that supports data capture, analysis and timely performance feedback against key measures of the process and outcomes of care for up to 20 hospitals. In each hospital data are captured by abstracting from paper medical records by a single clerical assistant, a process that has previously proven successful ^99^. Electronic records are not used at scale in the Kenyan public sector ^100^.

**Table 1.** ‘If, then, because’ statements that explicate our specific expectations of the network as a form of intervention.

If	then	because
***Level 1 – Network effects on national and county actors and the interface between level 1 and level 2***		
The Network produces trusted reports on key indicators for the quality of neonatal care across hospitals that are meaningful to key national and county level actors including the professional associations ***and*** is able to fully engage them in discussing these reports	Actors in the national and county governments, senior hospital management teams and key professional institutions and opinion leaders will begin to mobilise their influence and resources to support, sustain and spread improvement efforts and become more appreciative of hospital teams’ local efforts to improve	These key sectoral actors will share and embrace the goal to improve neonatal care, trust the performance information, accept some accountability for success in achieving the goals and become motivated to employ their formal or informal power to take on roles as institutional entrepreneurs to provide support and effect change rather than accept the status quo.
Actors in the national and county governments, senior hospital management teams and key professional institutions and opinion leaders are engaged and mobilised in supporting hospitals medical and nursing team leaders	Hospitals medical and nursing team leaders will engage more fully in the network activities themselves and be willing to lead and undertake improvement work within their hospitals	The opinion of those in authority, their endorsement of goals and active support for improvement are important normative influences on medical and nursing team leaders helping reshape professional identities, a process reinforced by the recognition of such actors which can become a powerfully motivating non-financial incentive
***Level 2 – Network effects on hospitals’ medical and nursing leaders and the interface between level 2 and level 3***		
The Network activities comprising outreach and mentorship, six monthly peer to peer meetings, performance feedback, and leadership and management skills development are effectively delivered with full participation of hospitals’ clinical and nursing team leaders	Hospitals’ medical and nursing team leaders will have the capability to conduct the local leadership and management work needed for improvement including: i) clarifying and communicating goals, ii) reflecting on performance feedback, iii) advocacy locally for essential resources, iv) promotion of better intra and inter-professional teamwork and v) creation of an organisational climate on wards that accepts change and engages families in care	Local leaders embrace the shared vision and goals for improvement as consistent with their own values, trust the performance information, accept some accountability for achieving improvements, feel supported by the network as a community, identify with the expanded professional leadership and management roles it encompasses, and are motivated by the recognition of their mentors, peers and hospital colleagues
Hospitals’ medical and nursing team leaders learn key relational skills and effectively engage over time in the day-to-day local leadership and management work that is needed to create better functioning teams	Frontline health workers will embrace the improvement goals and individually and collectively engage in practice changes that deliver improvements that are feasible with the available resources	Frontline health workers’ motivation is enhanced by feeling their contribution is valued locally and across the network, trust within teams who are now ’pulling together’ is increased, there is a renewed professional desire to provide quality care that aligns with personal values and accountability for improving newborn outcomes and families’ experiences becomes part of their identity
***Level 3 – Network effects on frontline workers and the interface between level 3 and families***		
The Network fosters team development, co-design and introduction of better job aides for frontline workers together with appropriate clinical and technical training and helps promote adoption of essential technologies	Despite some persistent resource challenges frontline health workers individually and collectively will have the basic skills, tools, resources and relationships to deliver essential forms of care to sick newborns over the many days and shift changes involved in inpatient stays	The training, job aides and additional technologies enhance their capability and opportunity to improve the quality of care while greater ownership of co-designed tools further enhances confidence, efficiency and self-efficacy, that with greater trust and recognition strengthen motivation and professional self-esteem
The frontline health workers embrace local team goals and professional norms that now emphasise providing comfort and support to babies’ mothers and family members	Mothers and family members will more fully participate in care, this will enable more effective breast milk feeding, promote babies’ growth, and help strengthen bonding between the parents and baby and relationships between families and staff	Staff will reconnect with values around caring so mothers and family members will feel more comfortable and confident on the ward and be more able to breastfeed and provide care, these better family relationships will further enhance workers’ satisfaction

## Intervention delivery

**Figure 5.**
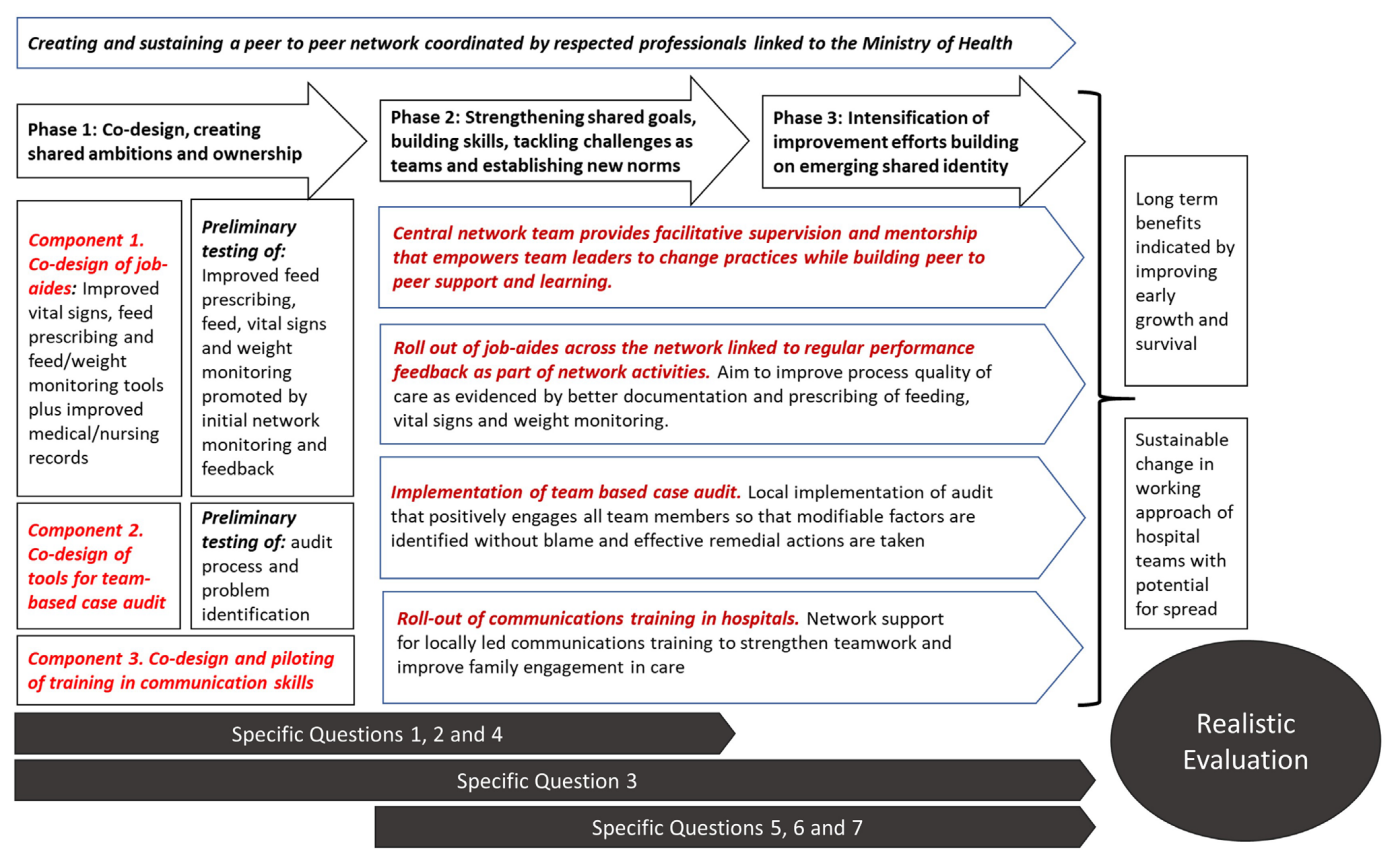
A simple representation of the activities planned as part of the network intervention outlining how we consider different phases within the overall approach. Intervention Phase 1 specific elements: **1a –** Co-design, piloting and revision of job aides and newborn unit (NBU) mortality audit tools. **1b –** Development and piloting of a short communications skills training for hospitals’ nursing leaders. Intervention Phase 2 specific elements: **2a –** Improve adoption of and adherence to Ministry of Health guidelines for inpatient neonatal care supported by use of finalised job aides and audit tools and provide regular feedback on performance at all levels of the health system. **2b –** Improve team-working and communications through training, peer support and network participation for hospitals’ NBU team leaders. Intervention Phase 3 specific elements: **3 –** Continuous network participation at all levels of the health system with a focus to improve attention to addressing challenges that worsen neonatal survival and adequate post-natal weight gain in vulnerable babies.

The network strategy we outline is neither feasibly or sensibly delivered as a ‘big bang’ intervention. The relationships and processes on which it is based will take time to establish and mature. These temporal considerations lead us to propose that the intervention, in the form of network activities, is structured in phases that are outlined in Figure 5. Phase 1 is dominated by developing the initial linkages across stakeholders and with NBU leaders building a shared vision and set of ambitions so that key personnel feel a sense of ownership of the change efforts. A key part of this phase is work to co-design tools and intervention components so that they facilitate or streamline work processes and help develop the team-based, trusting relationships that will be needed while simultaneously helping to embed shared aspirations for better care across all frontline workers. Four specific areas will be developed in this early phase that will be strengthened and sustained through subsequent phases. These include: i) work with hospitals to improve the quality of routine clinical information and the feedback mechanisms to be used to support improvement efforts at the national, senior hospital management and NBU team leader levels; ii) co-design of improved nursing charts to address the major problem of poor monitoring ^101^; iii) co-design of improved case review (audit) tools and processes that enable teams to engage positively and identify modifiable factors they can address to improve care locally; and iv) development of a communications skills training approach aimed at improving trust within teams and relationships between staff and families.

Subsequent phases build from these foundations to strengthen communication and relationships across the network, including use of mentorship and peer to peer discussions to further develop the skills and shared purpose of those at all levels and address practical issues of improving day-to-day clinical NBU care. Feedback that recognises and celebrates positive change and sharing stories of such change across the network through online and face-to-face exchanges will, it is hoped, provide momentum and encouragement to those acting as champions at levels 1, 2 and 3 who we think of more formally as institutional entrepreneurs doing identity work.

## Evaluation study design

We have outlined our efforts to develop a large-scale change intervention strategy targeting NBU in multiple Kenyan hospitals that is based on our understanding of context, prior research and what to us are relevant theories. As researchers tackle improvement challenges at increasing scale, moving beyond specific clinical guidelines or care pathways, greater attention is being paid to ‘systems thinking’ and ‘complexity theory’ ^59, 102^. These perspectives accept that interventions and contexts interact dynamically in ways that are not entirely predictable and seem particularly appropriate when changes in the behaviour of actors at multiple levels of the health system are needed. This does, however, mean that in designing an intervention strategy we must allow the process of intervention to remain somewhat flexible as ‘complex adaptive systems such as health care organizations and communities cannot be specified and managed in detail’ ^103^.

In such circumstances the investigational approach may also need to remain flexible, within certain bounds. In experimental approaches the aim is tight control or standardisation of the intervention to preserve internal validity. Where this is not possible some have suggested experimental designs can incorporate flexibility in delivering components of the intervention provided that there is sufficient standardisation of the anticipated processes through which they are expected to work ^104, 105^. When it may be hard to standardise intervention strategies, either because they are not fully elucidated or because they are potentially numerous and interacting, then our evaluation design may have to be optimised to advance simultaneously our understanding of mechanisms, assess important outcomes and examine the links between the two. Our aim in intervention and evaluation design is therefore to focus on maximizing learning and research rigour.

Employing a clinical network as an intervention poses a number of specific evaluation challenges. The use of an experimental design that relies on use of randomisation and controls is problematic for a number of reasons. Here we briefly highlight some key issues. First, the unit of intervention is the network itself even if it comprises multiple organisations or facilities. In this sense the direct comparator should be absence of a network, reducing comparison to one intervention and one control observation which would represent a weak experimental design even if such a study was feasible. Some might consider facilities to be the unit of intervention, these then might be randomly allocated to participation in a network or as ‘controls’. We have previously discussed how randomisation may not achieve equivalence or balance in such a situation if only relatively small numbers of complex organisations such as hospitals can be included for reasons of feasibility ^106^. Amongst these is that the list of known and unknown factors (confounders) that may influence a hospital’s response to intervention is potentially very long and time-varying ^106^. For example, in Kenya these may extend from change in political or hospital leadership to localised strikes to distinct differences in disease epidemiology ^107, 108^. More specific to the LMIC context may be lack of data from control settings needed to evaluate intervention effects on quality or outcomes of care ^109, 110^. To gain such data researchers may have to create and embed new data systems which themselves become a form of intervention ^111^. Denying ‘control’ hospitals access to information that may help them improve poses clear ethical challenges. For multiple reasons, therefore, examining the effects of networks might best be conducted using longitudinal study designs without controls based on a programme theory while using both quantitative and qualitative strategies to examine effects linked to a plausibility framework ^112^. These evaluation designs may take the form of ‘in-depth, mixed-method case studies that pay attention to interconnectedness and incorporate an understanding of how systems come together as a whole from different perspectives’ ^59^ and it is this form of evaluation we propose would be most useful to determine the effects of our network intervention and how it might produce both intended and unexpected effects.

### Our primary research question

How can a complex intervention comprising the development and sustained implementation of a multi-professional network (the change strategy) improve the quality of neonatal care and overall risk adjusted mortality in Kenyan hospitals and what transferable lessons can be identified that help advance programme theory to support future large-scale change interventions?

In tackling this overarching question, we will address a set of questions within sub-studies that advance our understanding of specific change efforts and effects.

1. Does co-design of job aides, audit tools and communications skills training improve their adoption and the quality of medical and nursing care?
2. Which common factors need to be modified to improve inpatient neonatal care and outcomes?
3. How do important process of care quality indicators change in response to feedback and how do these indicators and mortality change over the period of network participation?
4. What is the optimum approach for risk adjustment so that variations in case-mix and case-severity can be accounted for when evaluating temporal trends in inpatient neonatal mortality?
5. How do senior doctors and nurses’ social ties evolve within the network, influenced by face to face and online interaction, and influence the performance of frontline staff?
6. How does information being produced by the network reach policy makers and other influential stakeholders and how might performance data be influential in fostering network growth and wider health system improvement?
7. How can better performance measurement be integrated into national information systems to sustain improvement and future national networks?

In Phase 1 and extending into Phase 2 we will examine Questions 1 and 2. We will use data from a pre-intervention period and Phase 1 to develop and then deliver performance feedback on agreed quality indicators in Phase 2 and validate prognostic models for mortality (addressing Question 4) that can be used in future risk adjusted analyses of programme effects on neonatal outcomes. Across the phases of the intervention we will roll out the job aides, audit tools and communications training and link this with facilitative supervision, mentorship and peer learning with the aim of improving day-to-day clinical NBU care. Our aim is to intensify the focus on mortality reduction in Phase 3 using continued feedback and sharing stories of effective change across the MPN and with senior national level stakeholders. This will provide momentum and encouragement to key individuals at all organisational levels to strengthen change efforts. Data will be collected as the intervention matures through Phases 1, 2 and 3 to examine the evolution of the MPN and its effects (tackling specific Questions 3, 5 and 6) and explore how performance measurement might be integrated into the national health information system (addressing specific Question 7). Data on changes in quality indicators and mortality from across the pre-intervention period and Phases 1 to 3 together with qualitative data from across sub-studies will be employed in the Realistic Evaluation. Further information on the proposed conduct of sub-studies is provided in Table 2.

**Table 2.** Specific questions - sub-study designs and methods.

Study design and methodology	Study site; study populations; sampling procedures; and data collection procedures
***1. Does co-design of job aides and communications skills training support improve adoption of innovations and the quality of*** ***medical and nursing care***	
Rapid cycle co-design meetings with hospital staff of job aides (e.g. to support better documentation of vital signs observations and feed prescribing), structured team based case review (TCR audit) tools and to adapt an existing communications training will be used with pilot testing in these sites for an initial six months. ***(Completed)*** ***Currently -*** Introduction of new job aides and TCR audit tools is being progressively extended to MPN hospitals in the closing part of Phase 1 in preparation for Phase 2 when feedback on key indicators and MPN peer engagement will be used to promote their use. The adoption of job aides and their early effects on process measures of quality of care will be evaluated in Phase 2 to inform continued efforts to promote adoption.	*Study sites:* We will seek 2 - 3 MPN hospitals as volunteers for the rapid-cycle co-design activities prior to progressive pilot implementation in the remaining 8 - 9 hospitals. *Study populations:* Nurses and medical staff will be identified by hospitals to work with researchers to co-design job-aides and mortality audit tools and subsequently implement these. For piloting adoption of job aides, medical records of NBU admissions in the pilot period will be sampled. *Procedures:* Hospital records for NBU admissions for whom a co-designed job aide should be used / completed will be used to ascertain adoption and use these pilot studies data on up to 30 cases in 2-3 hospitals, allowing further redesign of job aides as needed. Notes and results of group meetings to co-design TCR audit tools and communications training will be used to assess the design process and improve tools prior to wider scale implementation as part of a Human Centred Design approach.
***2. Description of common modifiable factors in providing effective feeding to sick babies.***	
We will promote use of the structured neonatal TCR audit approach co-designed in Phase 1 across the MPN during Phase 2 and 3 to review late deaths occurring on the NBU (those ≥ 3 days post-natal age) to identify modifiable factors occurring during the NBU stay. TCR audit reports will be collated as part of MPN activities and aggregate reports generated on modifiable factors from across the 11 hospitals. Pooled data will be used to describe the nature and frequency of modifiable factors across hospitals.	*Study sites:* This work will include all 11 MPN hospitals. *Study* *populations:* Hospitals will be asked to review late post-natal deaths (those ≥ 3 days post-natal age at time of death) each month (noting that the Ministry of Health expects all neonatal deaths to be reviewed). *Data collection procedure:* Deidentified data will be collected from hospitals’ structured neonatal TCR audit reports. We aim to collect data on 250 cases as the basis for a report summarizing findings from all hospitals.
***3. How do important process of care quality indicators and mortality change over the period of MPN participation***	
Building on Phase 1 and the earlier establishment of the information system we will track and develop feedback systems on quality of practices such as feed prescribing, monitoring (e.g. assessment with pulse oximetry) and mortality outcomes and progressively use this to focus hospital teams’ attention on their performance in providing quality care and achieving good survival outcomes. We will aim to further drive local learning on how to improve care through online and MPN meetings between hospitals and promote the use of the information from locally conducted neonatal TCR audits that identify factors that can be modified to improve outcomes.	*Study sites:* This work will include all 11 MPN hospitals. *Study* *populations & sampling:* We will examine medical records for all NBU admissions in Phases 1, 2 and 3 and especially those meeting criteria of being a vulnerable baby (either preterm birth (<37 weeks gestation) or low birth weight (<2500g). *Data collection procedure:* All NBU records from which routine de-identified data are **currently being captured** and we will use the quality indicators developed (e.g. on feeding practices and correct antibiotic use) to provide monthly mortality reports and three monthly summary reports on quality of care indicators. We will track the change in aggregate and hospital specific performance and mortality outcomes over time.
***4. What is the optimum approach for risk adjustment of neonatal mortality so that variations in case-mix and case-severity can*** ***be accounted for when evaluating temporal trends in inpatient neonatal mortality***	
We will build on prior work undertaken in a single Kenyan hospital to develop two candidate prognostic scores for neonatal mortality and **use data already captured** to undertake external validation and improvement of these modelling approaches. The aim will be to develop a prognostic scoring approach that can be applied at individual patient level that enables us to undertake risk adjustment when tracking the long-term trends in survival outcomes in response to the MPN intervention.	*Study sites:* This work will include all 11 MPN hospitals. *Study* *populations & sampling:* We will use **existing data on over 30,000** **NBU** to validate / revise a preferred prognostic model and then use this model to provide risk-adjusted estimates of mortality each month for the 11 sites for a total of 36 months across Phases 1, 2 and 3. These data will enable us to say with greater certainty whether NBU mortality rates are declining over the period of intervention and potentially in which sub-populations.
***5. How do senior doctors and nurses’ social ties influence the performance of frontline staff and evolve within the MPN influenced*** ***by face to face and online interaction***	
Building on work to develop and implement the communications training we will progress to examine the relationships between medical and nursing staff within and between hospitals. This work will be informed by conduct of a realist review of social MPN analyses conducted on hospital staff and based on this proceed to empiric work involving in-depth interviews (IDIs) with senior hospital staff, small group discussions with frontline workers and family members, and episodes of non-participant observation of everyday practices. The aims of this data collection will be to explore changes in how MPN participants perceive their roles, their teamwork, the practices they employ to improve neonatal and family centred care and how these may all be mediated by the creation or strengthening of social ties resulting from MPN participation.	*Study sites:* Medical and nursing leads from all 11 MPN hospitals’ NBUs will be eligible for IDI and four hospitals representing maximum variation in performance on quality indicators being tracked will be identified for further IDI and small group discussions. In these four purposefully selected hospitals, non- participant observation will be conducted and front-line staff and family members will be invited for small group discussions based on convenience sampling but employing *inclusion criteria* to ensure diversity in respondents (e.g. based on age, qualification (for staff), gender and education (for family members) The aim is to conduct two small group discussions with staff (total four to six people) and families per site.
***6. How does information being produced by the MPN reach senior advisory and advocacy group members and how may it be*** ***influential in fostering MPN growth and wider health system improvement***	
The MoH Technical Group of Experts is expected to meet three times monthly in Phases 2 and 3. In the last 3 months of Phase 3 IDI will be conducted with group members and key wider stakeholders. We will explore their opinions on the value of the information being created by the MPN, their experience of their work as a group, and how such information might be used in the wider health system context to promote improved NBU care. Activities or reports have been of value in helping support decision making.	This work will focus on the national level and at the level of county administrations where these have hospitals included in the MPN. *In* *Phase 3* IDI will be conducted with members of the MoH Technical Group of Experts (n = 6 - 8), health care policy makers, key donor partner personnel (e.g. UNICEF, WHO) and selected senior county and hospital managers (total n = 6 - 10). *Sampling procedures:* Sampling will be purposeful with a snowballing approach used to identify relevant interviewees in national or county government, hospital management or donor organisations.
***7. How can better performance measurement be integrated into national information systems to sustain improvement***	
We have conducted with the MoH (Informatics Division) a careful appraisal of existing neonatal data collection approaches within the national DHIS2 system revealing considerable weaknesses ^110^. We will draw on prior work ^38^ to use consensus development methods to develop a core set of preferred neonatal quality and outcome indicators for use at national level. We will follow this with use of human centred co-design approaches to improve the process of neonatal data capture and the DHIS2 tools that support it to enable long-term, large scale tracking of neonatal care and outcomes embedded in the national DHIS2.	This work will be conducted at the national level with the Ministry of Health and its Neonatal and Child Health Unit and its Informatics Division. A stakeholder meeting, linking to one of the proposed MPN meetings, will be held to review and suggest revisions to the current information procedures and tools. Draft quality and outcome indicators developed will be refined and agreed with key stakeholders though the Ministry of Health Technical Group of Experts. Based on this process we will revise existing paper tools and online DHIS2 data capture tools employing co-design workshops and ‘walk-throughs’ with health records information officers and clinicians to develop minimal viable products (MVPs) for initial testing. We will progress these MVPs so they can be incorporated into the DHIS2 system and potentially extend this by enabling distributed data capture through mobile applications.

TCR, team based case review; MPN, multi-professional network; NBU, newborn unit; IDI, in-depth interview; MoH, Ministry of Health; MVP, minimal viable product.

### Quantitative analyses and sample size

Data from across the phases will be used to develop time-series models interrogating the rate of improvement in process indicators and health outcomes over time and as the network intervention progresses. Data will be collected from the national information system (DHIS2) on at least 30 hospitals not in the network over the same time period. These data will provide a frame of reference that can help us interpret whether any changes in intervention hospitals are plausibly related to intervention or linked to wider secular changes in hospital neonatal mortality in Kenya.

Existing pooled data from 11 hospitals we initially plan to work with and for which we have pre-intervention data indicate we can expect denominators of approximately 1200 babies of any weight and 400 low-birthweight (LBW) babies to be admitted to these NBUs per month. Such denominators would allow for specific monthly performance indicators across the network to be estimated with 95% confidence intervals around the most conservative proportion of 50% of ± 5% and ± 3% for LBW and all babies, respectively. These sample sizes are sufficient for detecting meaningful changes in performance and health outcomes to inform our plausibility analysis and later realistic evaluation. For example, in pooled data across sites inpatient mortality at baseline is currently estimated to be 10% +/- 1%. In an end-line period of three months if crude mortality in the same hospitals is 8% or lower, this would likely represent a significant change. Using appropriate, individual risk adjustment in time series analyses will improve the rigour of these analyses. These sample sizes will also be sufficient for detecting clinically important changes in quality of care indicators as we have demonstrated in previous paediatric work ^36, 37, 113^.

A large body of qualitative data will be collected linked to the specific questions we outline above and that enable us to examine elements of the starting programme theory ( Table 2). Data from across all the sub-studies will be analysed using the realist logic of analysis as set out by Pawson and Tilley ^10^. The goal of the data analysis is to further develop the programme theory ( Figure 5) so that it will provide more complete explanations for how the MPN intervention results in changes of the process indicators and health outcomes. This analysis will pay careful attention to the changes in behaviour of actors at multiple levels of the health system that are hypothesised to be necessary to effect large scale change and in keeping with recommendations to conduct in-depth, mixed-method case studies ^59^. To further complement this work and provide important contextual data we will collate information on other programmes being implemented in all Kenyan hospitals and those within the MPN that may influence their neonatal care as well as any major changes in national or county government policies. In addition, the team will use reflective team meetings to carefully document actual network activities taking note of any changes that occur during the process of intervention and recording their reflections on the reasons for these. Our analyses will allow us to explore unintended and potentially emergent consequences of the intervention. For example, we can pay particular attention to how Kenya’s response to the COVID-19 pandemic influences the provision of neonatal care in the short and long-term. We will use this process of critical enquiry to advance our programme theory of what worked, why and under what conditions to improve NBU care ^10^. We will present findings in the form of context-mechanism-outcome configurations that will inform our updating of the programme theory which we will use to guide the design of new large-scale improvement programmes.

## Conclusion

In LMIC contexts there has been growing interest in embedded research ^114^ and long-term learning sites to help understand large scale change ^115^, although researcher-led large-scale change interventions are perhaps less common ^42, 116, 117^. Others focused on large scale change in LMIC have focused on the politics and governance of such change or employed Realistic Evaluation as a central strategy to explore hospitals’ management ^118, 119^. Work in the latter arena has contributed significantly to thinking on the effects of hospital management on improvement and change processes ^120, 121^ and on the evolution of specific service delivery platforms ^97^. In HIC the UK NHS Institute for Innovation and Improvement presents a more practical guide to large scale change interventions ^7^ and there have been efforts to learn from the UK’s Collaborations for Leadership in Applied Health Research and Care and geographically widespread service improvement interventions ^122, 123^. Often, however, research is conducted in tandem with an existing intervention or used to provide ex post explanations for programme success or failure. Here, we attempt to provide a programme theory, propose to undertake the intervention it informs and subsequently evaluate its progress. The longer-term aim will be to revise the programme theory as part of a process of creating knowledge that is transferable and may help optimise future LMIC interventions.

## Data availability

No data are associated with this article.
