# Supplementary material for: Programme theory and linked intervention strategy for large-scale change to improve hospital care in a low and middle-income country - A Study Pre-Protocol
Source: Wellcome Open Res. 2020 Dec 18;5:265. Originally published 2020 Nov 5. [Version 2] doi: 10.12688/wellcomeopenres.16379.2 (PMC7684682; doi:10.12688/wellcomeopenres.16379.2)
Supplement: Peer review history

# Peer review: Reviewer response for version 2

**DOI:** 10.21956/wellcomeopenres.18167.r41877

**Published:** 2021-01-13

**Authors:** Ferdinand C Mukumbang

**Affiliations:** 1 School of Public Health, Faculty of Community and Health Sciences, University of the Western Cape, Cape Town, South Africa

I have had the opportunity to review the article again in light of the comments and responses of the authors and I am happy to "APPROVE" the article.

Is the study design appropriate for the research question?

Partly

Is the rationale for, and objectives of, the study clearly described?

Partly

Are sufficient details of the methods provided to allow replication by others?

No

Are the datasets clearly presented in a useable and accessible format?

Not applicable

Reviewer Expertise:

NA

I confirm that I have read this submission and believe that I have an appropriate level of expertise to confirm that it is of an acceptable scientific standard.

# Peer review: Reviewer response for version 1

**DOI:** 10.21956/wellcomeopenres.18016.r41309

**Published:** 2020-12-04

**Authors:** Janet Myers

**Affiliations:** 1 Division of Prevention Science, Department of Medicine, University of California, San Francisco, San Francisco, CA, USA

- Is the rationale for, and objectives of, the study clearly described?

The need to reduce neonatal mortality is clearly urgent which helps justify the rationale and the objectives for the study proposed.

 It might be nice to include a diagram or revise the figure or just add additional explanation to show how the network strategy and the things it fosters such as local institutional entrepreneurs can influence the proximal and distal outcomes in Figure 1. Generally, I could use a little more help in understanding how the intervention components described will impact the outcomes in Figure 1. I wonder if more explicitly and completely including the voices of the women and families served in the research design might help make this connection clearer.

 The study itself will be driven by 7 research questions. The work in questions 6 and 7 almost seems like a stand-alone dissemination study. This study actually seems like a life’s work with questions 1 through 5, so it could be better justified why questions 6 and 7 are included. I definitely see the importance of them, but there is a lot going on here. Also, they could benefit from the inclusion of consumer voices (sorry to be a broken record on this, but it’s pretty critical with women and children key pops).

  

- Is the study design appropriate for the research question?

The use of Realistic Evaluation is justified because of the self-reflective position of the evaluators.

 The use of in-depth, mixed methods with a heavy emphasis on qualitative inquiry is appropriate to the design; in a sense this is a large case-study of a network intervention and so the study design is appropriate.

  

- Are sufficient details of the methods provided to allow replication by others?

This nature of the intervention and the methods chosen to evaluate it are in this case unique and highly specific. For this reason, replication is not likely. However, the detailed case for the theoretical underpinnings of the intervention should facilitate understanding of the mechanisms by which change was or was not achieved, and the methods for measuring this change are clear. For this reason, others seeking to replicate pieces of the intention or even an adapted version of it should be able to glean sufficient detail to allow useful comparison if not replication. Table 2 and the narrative surrounding it do a good job of describing the methods in detail.

 Throughout there are opportunities to include consumers more than is described – for example, they could help in designing job aids and care strategies.

 The quantitative outcome evaluation is well described. More detail could be provided on some of the process measures. For example, how the completion and use of job aids will be assessed, how adoption will be ascertained, how the results of group meetings will be assessed and how tools and improvements will be measured. There is mention that these things will be done, but not how they will be assessed in detail.

  

- Are the datasets clearly presented in a useable and accessible format?

The quantitative data set is well described and the investigators have experience using it so it seems highly feasible and usable. The qualitative data collection is less well described as is any assessment of process indicators like how well a job aid did or didn’t work, how many people participate in the planned activities, etc. Table 2 does a nice job with the methods overview, but additional detail about how the qualitative data will be collected and used alongside the quantitative data would be helpful. 

**Minor edits suggested:**

 Two adjacent instances of “However” in the abstract.

 In Table 2, only Question 2 is framed as a statement, not a question, which seems odd.

 A caveat: This article relies heavily on bodies of theory I’m not familiar with (as I am basically a US-based implementation scientist); however, the justification for the selection of the theories such as network theory are good and the overall logic for the intervention is sound.

Is the study design appropriate for the research question?

Yes

Is the rationale for, and objectives of, the study clearly described?

Yes

Are sufficient details of the methods provided to allow replication by others?

Yes

Are the datasets clearly presented in a useable and accessible format?

Partly

Reviewer Expertise:

Implementation science, health services research, program evaluation

I confirm that I have read this submission and believe that I have an appropriate level of expertise to confirm that it is of an acceptable scientific standard.

# Response

**Published:** 2020-12-05

**Authors:** Mike English

**Affiliations:** University of Oxford and KEMRI-Wellcome Programme, UK

Thank you for carefully considering our report and for offering suggestions for revision. We have tried to make particular changes to the text (in section titled - An initial programme theory for an intervention targeting improvements in care on Kenyan NBU) to address the issue of how our intervention approach is linked to the quality of care issues identified in the driver diagram (Figure 1). Part of the challenge of what we are trying to do is that it is hard to isolate how a particular feature of the intervention strategy impacts on a particular driver. In fact, one of the ideas we hope is conveyed is that it may be a mistake to try and tackle very specific quality problems in isolation. Adapting Figure 1 or adding a table to illustrate how the intervention elements impact the drivers directly is somewhat contrary to a central tenet that we are dealing with complex systems. We did consider trying to develop causal loop diagrams that might be better suited to illustrating the complexity of interconnections but these soon seemed to become unmanagably complex. So we have tried to address this issue, partly, by providing additional text that points to how different intervention elements may work together to address the drivers of poor quality care.

 We also acknowledge that objectives 6 and 7 are themselves fairly major research topics and therefore add to the ambition of this work. In this pre-protocol we wanted to set out our intent in advance of the work itself in an effort to be transparent about our aims. We hope that we can achieve them and by laying out the intent now we will owe people an explanation of why we did not achieve them if this turns out to be the case.

 It is an excellent point that the family members, especially the mothers are also key stakeholders and partners in our network. This is an area we are beginning to work on more actively but did not feel we had the capacity to include in a more formal part of the intervention planning at this stage, although we intend the communications training to have as a strong focus staff-family relationships that we hope will empower families as partners in care.

 We agree with the comment that what we are proposing may not be replicable directly in another context. As suggested in your comment the real purpose of publishing this report is to demonstrate how we have developed our ideas in the hope that this example is helpful to others also attempting to employ theory more explicitly in their strategies to achieve change.

 We hope we have addressed the minor comments in our revisions.

# Peer review: Reviewer response for version 1

**DOI:** 10.21956/wellcomeopenres.18016.r41310

**Published:** 2020-11-23

**Authors:** Ferdinand C Mukumbang

**Affiliations:** 1 School of Public Health, Faculty of Community and Health Sciences, University of the Western Cape, Cape Town, South Africa

Thank you for the opportunity to review such a rich paper.

 I would like to suggest a complete rewrite of the abstract. I found it very hard to follow. The ideas did not in my opinion flow seamlessly and I struggled to make sense of what the authors are trying to achieve from the information provided in the abstract. Some of the sentences there could also be simplified.

 The authors should offer a simple definition of program theory to help unfamiliar readers.

 One of my major concerns is that the aim of the study is not clear. I am not really sure what the authors set out to achieve in this paper. They outlined a bunch of things that they sought to achieve but it is unclear what the aim of the paper is.

 It would have been more appropriate to see how the authors embed their data collection procedures in the realist evaluation research cycle. This would provide better clarity on how they obtained their initial programme theory and how they intend to refine this programme theory to explain the process of large scale transformation.

Is the study design appropriate for the research question?

Partly

Is the rationale for, and objectives of, the study clearly described?

Partly

Are sufficient details of the methods provided to allow replication by others?

No

Are the datasets clearly presented in a useable and accessible format?

Not applicable

Reviewer Expertise:

Health policy and systems research; Critical realist-informed research including realist evaluation and realist reviews; Implementation sciences; Theory-driven evaluations.

I confirm that I have read this submission and believe that I have an appropriate level of expertise to state that I do not consider it to be of an acceptable scientific standard, for reasons outlined above.

# Response

**Published:** 2020-12-05

**Authors:** Mike English

**Affiliations:** University of Oxford and KEMRI-Wellcome Programme, UK

Thank you very much for your review and the considered comments you made to suggest improvements. We have, we hope, addressed your concerns in the revised manuscript now submitted. Specifically, we have rewritten the abstract and introduction. We hope the purpose or aims of the manuscript are now clearer and that the struture of our report is also better signposted and justified. We have also indicated how we feel our work is aligned with the Realist Evaluation cycle, noting that it is largely an example of the first phase of this cycle to develop an initial programme theory. However, we note that in doing this we drew on a considerable body of prior research and experience in the specific context in which we propose to intervene.

 We hope the revised text is an improvement.

# Peer review: Reviewer response for version 1

**DOI:** 10.21956/wellcomeopenres.18016.r41307

**Published:** 2020-11-23

**Authors:** Amy Gray

**Affiliations:** 1 Department of Paediatrics, University of Melbourne, Melbourne, Vic, Australia

The authors have published a study pre-protocol which is an interesting read, that for others provides a great breadth of references to theories relevant to improving care and health systems. In particular they focus on improving neonatal care in Kenya which is appropriate given the complexity of neonatal care and the need for inter-professional cooperation if it is to succeed at the hospital level - and therefore warrants this attention.

 The authors provide a programme theory which I believe appropriately draws on different theories or frameworks including complexity theory, networks as vehicles for change, distributed leadership, trust, and self determination alongside governance and notions of feedback.

 This is a complex and challenging piece to write given the depth that could be explored in each of these areas of work alone, let alone together.

 The description of the planned phased evaluation is appropriate in relation to the questions to be answered and in the use of realist evaluation.

 The clear intervention they have identified is a multi-professional network - which itself rests on a relatively small central team which interacts with other levels of hospital networks. It is less clear to me how they have chosen specific activities that this team will undertake including outreach/mentorship, peer-to-peer mentoring, feedback, coaching and communications training (nursing). Some of these are hinted at within the relevant theories for frameworks. But as activities many are poorly articulated in the current literature as to what is done when these activities are undertaken. For example, coaching is an approach which could apply equally to leadership as it does to communications training. Nor it is quite clear why if the focus is on an inter-professional network, why different members of this network (eg doctors and nurses) have been singled out for different activities, when the aim is to work together. Having stated this, the co-design of many of these components seems critical - thus allowing for future clarity in their definition.

 This is an ambitious undertaking, but it is the type of research which is needed if we are to achieve complex health reform at scale.

Is the study design appropriate for the research question?

Yes

Is the rationale for, and objectives of, the study clearly described?

Yes

Are sufficient details of the methods provided to allow replication by others?

Partly

Are the datasets clearly presented in a useable and accessible format?

Not applicable

Reviewer Expertise:

Quality of care, Education, Child Health, Global Health, Implementation research.

I confirm that I have read this submission and believe that I have an appropriate level of expertise to confirm that it is of an acceptable scientific standard.

# Response

**Published:** 2020-12-05

**Authors:** Mike English

**Affiliations:** University of Oxford and KEMRI-Wellcome Programme, UK

Thank you for taking the time to review this article and for your comments. We have tried to address the issue of why the particular intervention components were selected and why they might have specific target groups in the revised manuscript. However, we are also conscious of the length of the piece already and so tried to keep these explanations brief. We also recognise, and mention this in the section on intervention design, that the intervention itself will likely evolve. For example, we indicate that initially we will target communications training at senior nurses because prior work has identified major staff-family communication problems. However, as this work progresses and the multi-professional network evolves intervention components such as communications training may well extend beyond the original target group in response to demand or direction from our partners in the network.

# Peer review: Reviewer response for version 1

**DOI:** 10.21956/wellcomeopenres.18016.r41308

**Published:** 2020-11-06

**Authors:** Hamish Graham

**Affiliations:** 1 Centre for International Child Health, MCRI, Royal Children's Hospital, University of Melbourne, Parkville, Vic, Australia

This paper is a pre-protocol paper addressing the context and theory underpinning potential large-scale interventions for improving neonatal care in Kenya.

 The authors begin by describing the challenges facing neonatal care in Kenya, with a focus on mid-level (district/country) hospitals and healthcare delivery by nurses and junior medical staff.

 Drawing on their experience with professional networks, the authors explore the theoretical mechanisms through which networks can contribute towards large-scale change. While I am not familiar with all the theoretical influences here, I am impressed by the breadth of theories considered, and the coherent presentation of how they may contribute to an overall program theory and intervention design.

 The authors then present a program theory depicting the use of a 'multi-professional network' as an intervention to drive large-scale improvement in neonatal quality of care. The program theory clearly emphasizes the importance of relationship, ranging from micro (e.g. parent-nurse relationships), meso (e.g. professional relationships within and between facilities), to macro (e.g. government, management and professional institutions). In describing these relationships and the proposed particular interventions (e.g. job aids, mentoring program, audit activities), the authors clearly have a good understanding of the complexity of patient care and how a 'network' intervention might work.

 The authors then lead into an excellent discussion about what evaluation approach is appropriate, before articulating the research questions. (Though I wonder if the primary question is actually "how can...", rather than just "can...".)

 This is an excellent study protocol (or pre-protocol), which in itself will be a great resource to others seeking to design and evaluate programs for large-scale health service improvement.

Is the study design appropriate for the research question?

Yes

Is the rationale for, and objectives of, the study clearly described?

Yes

Are sufficient details of the methods provided to allow replication by others?

Yes

Are the datasets clearly presented in a useable and accessible format?

Not applicable

Reviewer Expertise:

Child health, quality improvement,

I confirm that I have read this submission and believe that I have an appropriate level of expertise to confirm that it is of an acceptable scientific standard.

# Response

**Published:** 2020-12-05

**Authors:** Mike English

**Affiliations:** University of Oxford and KEMRI-Wellcome Programme, UK

Thank you for taking the time to review this article and your positive comments.
